# Colloidal Dispersions of Gramicidin D in Water: Preparation, Characterization, and Differential Cytotoxicity

**DOI:** 10.1021/acsomega.4c11133

**Published:** 2025-02-24

**Authors:** Ricardo Márcio-e-Silva, Bianca R. Bazan, Rodrigo T. Ribeiro, Sarah N. C. Gimenes, Bianca C. L. F. Távora, Eliana L. Faquim-Mauro, Ana M. Carmona-Ribeiro

**Affiliations:** 1 Biocolloids Laboratory, Departamento de Bioquímica, Instituto de Química, Universidade de São Paulo, Avenida Professor Lineu Prestes, 748, Butantan, São Paulo, SP 05508-000, Brazil; 2 Immunopathology Laboratory, Butantan Institute, Av. Vital Brasil, 1500, São Paulo 05503-900, Brazil

## Abstract

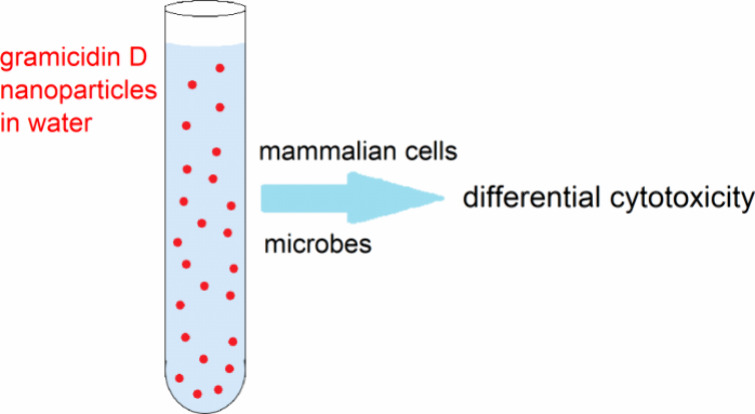

Gramicidin D (Gr) is a natural mixture of channel peptides A–C with minor differences in chemical structure, which are able to span cell membranes as dimers. These Gr channels allow single-file diffusion of cations, thereby disrupting the usual ionic balance in biological cells and inducing cell lysis. The microbicidal activity of Gr using different carriers such as bilayer vesicles or bilayer disks, supported bilayers on silica, or polystyrene nanoparticles has been described. Gr antimicrobial activity was found to depend strongly on its formulation. Preliminary description of self-assembled Gr nanoparticles (Gr NPs) by our group showed a superior antimicrobial performance for these Gr self-assembled nanospheres. In this work, we further characterize Gr colloidal dispersions in aqueous solution over a range of micromolar concentrations from turbidimetry, obedience to the Rayleigh law for light scattered by NPs smaller than the wavelength of the incident light, dynamic light scattering to ascertain the reproducibility of physical characteristics of Gr NPs, and effects of Gr NPs on the cell viability of five different mammalian cell lines in culture over a micromolar range of Gr concentrations (0.5–5.0 μM). Thereby, the differential cytotoxicity of Gr NPs is inferred from the comparison between effects on microbial cell viability and mammalian cell viability. The results suggest that the simple and efficacious formulation of Gr NPs obtained directly from Gr self-assembly in aqueous solution deserves to be further exploited, aiming at systemic biomedical uses of Gr in vivo against infectious diseases and cancers.

## Introduction

Biomimetic systems have evolved to mimic assemblies of natural biomolecules and represent a valuable strategy to obtain artificial but functional supramolecular assemblies.^1^ Natural or synthetic peptides have been explored to create a whole new set of applications in drug delivery, materials science, molecular biology, and bionanotechnology.^2^ Antimicrobial peptides can assemble driven by inter- and intramolecular interactions yielding nanospheres, nanofibers, nets, sheets, nanotubes, or hydrogels.^3^ Cyclic peptides conjugated to polymers self-assembled as peptide–polymer nanotubes able to insert in membranes, act as ion channels, deliver anticancer drugs or genetic materials, or provide applications as antivirals.^4^ Sophisticated techniques such as super-resolution microscopy with modern image analysis, small-angle neutron scattering, and solid-state nuclear magnetic resonance have allowed characterization of nanostructures from short peptides with strategic applications in biomedicine and nanotechnology as antimicrobials, anticancer agents, vehicles for controlled drug release, peptide bioelectronics, and responsive cell culture materials.^5^ In drug and vaccine delivery, self-assembled peptides represent a class of materials that can be tailored to drug targeting and vaccine design.^6−11^ For example, self-assembled dipeptide-based fluorescent nanoparticles have been developed as a platform for imaging cellular probes and targeted drug delivery chaperones.^12^ Self-assembled short peptides have indeed been called for innovative pharmaceutical applications.^13^ Against pathogenic microbes, self-assembling peptides have also been useful in the battle against antibiotic resistant strains.^14,15^

A peptide molecule in water solution exhibits a conformation of minimum free energy under equilibrium conditions. Physical interactions driving the self-assembly of peptides in water solution such as hydrogen bonding, π–π, cation−π, electrostatic, hydrophobic, and van der Waals interaction forces can be reasonably long-ranged and able to cooperatively yield energies equivalent to a weak covalent bond.^16^ These physical interactions acting cooperatively can yield a variety of nanostructures such as nanotubes, nanospheres, and nanofibers.^17^ Interesting biomimetic constructions have been described such as two-dimensional giant nanosheets obtained from the self-assembly of d/l-alternating cyclic peptides; one-dimensional self-assembly into amphiphilic nanotubes was followed by arrangements as tubular layers forming the giant nanosheets.^18^

There is a general trend in the literature to formulate antimicrobial peptides as assemblies able to overcome in vivo degradation.^8^ These assemblies can be self-assemblies^19,20^ or assemblies of modified peptides with added molecular moieties that are able to promote their assembly.^21^ Besides promoting protection against proteases, in certain instances, enhanced activity of the assembled peptides was observed.^19,22,23^ Furthermore, they offer antibiofilm applications important for hampering formation of microbes consortia on a variety of materials.^24−27^

Recently, the self-assembly of the channel peptide gramicidin D (Gr) as nanoparticles in water dispersions was reported for the first time.^19^ The Gr NPs in aqueous dispersion showed remarkable activity against *Candida albicans* but barely affected bacteria such as *P. aeruginosa*. As coatings deposited on either glass or polyethylene surfaces, Gr NPs in combination with cationic antimicrobial polymer were able to synergistically reduce the cell viability of the three quoted microbes by several logarithmic cycles yielding complete loss of cell viability.^26,27^ The mechanism of action was dual, with the cationic polymer combining with biopolymers from the microbial cell wall and facilitating Gr NPs’ interaction with the cell membrane for disruption of the cell ionic balance. Despite the transient character of the Gr/cationic polymer coatings, which were easily detached from the surfaces upon washing out, they may still find utility for providing biomedical devices with timely protection over a limited period of time.^27^ Comparing combinations of gramicidin/polymer with conventional antibiotics also having a broad spectrum of activity, there are important differences in their mechanisms of action. Antibiotics usually affect metabolic pathways in the pathogen, allowing the appearance of resistance via efflux pumps and other mechanisms for blockade of cell metabolic pathways.^28−31^ Gr/cationic polymer combinations act via the electrostatic interaction between the cationic polymer and the biopolymers of the cell wall, which causes cell wall disruption,^32^ thereby facilitating gramicidin access to the pathogen cell membrane.^19,26,32−37^ In the cell membrane, the diffusion of cations through the gramicidin channel disrupts the ionic balance of the cells, leading to cell lysis. Gramicidin is among the oldest lytic peptides reported but still considered in its infancy regarding further evaluation as an anticancer weapon.^38−41^

In this work, we further characterize Gr colloidal dispersions in aqueous solution over a range of Gr micromolar concentrations from turbidimetry, obedience to the Rayleigh law for light scattered by NPs smaller than the wavelength of the incident light, dynamic light scattering for ascertain reproducibility of physical characteristics of Gr NPs, and effects of Gr NPs on cell viability of five different mammalian cell lines in culture over a micromolar range of Gr concentrations (0.5–5.0 μM). Thereby, the differential cytotoxicity of Gr NPs is inferred from the comparison between effects on microbial cell viability and mammalian cell viability. The results suggest that the simple and efficacious formulation of Gr NPs obtained directly from Gr self-assembly in aqueous solution deserves to be further exploited, aiming at systemic biomedical uses.

## Experimental Section

### Materials

d-Glucose was obtained from Sigma (Steinheim, Germany). Gramicidin D (a peptide mixture consisting mostly of Gr A), ethanol, chloroform, and 2,2,2-trifluoroethanol (TFE) were purchased from Sigma-Aldrich (St Louis, Missouri, USA). HCl was obtained from Sigma (St. Louis, Missouri, USA).

### Preparation of Gramicidin Dispersions in Water

Gramicidin (Gr) stock solution at 5.0 mM Gr was used to prepare 2 mL of dispersions in ultrapure water to yield the desired final concentrations. The final TFE concentration was kept at 1% of the final volume of the water dispersions by first adding 0.02 mL of appropriate Gr solutions in TFE to the bottom of an assay tube and then dispensing 1.98 mL of pure water with an automatic pipet. Gr NPs prepared in 1% TFE and used at 0.1% TFE against the cells were not purified. For further studies in vivo to be performed in the future, TFE elimination might be achieved by dialysis and would be recommended.

### Determination of Physical Properties of Gr Dispersions in Water

Physical characteristics of Gr nanoparticles were determined by dynamic light scattering (DLS) such as the zeta-average diameter (Dz), zeta potential (ζ), polydispersity (*P*), and conductance (*G*) by using a Brookhaven apparatus (ZetaPlus Zeta Potential Analyzer, Brookhaven Instruments Corporation, Holtsville, New York, USA) equipped with a 677 nm laser. The principles of DLS were explained in detail beforehand.^42^ Briefly, the relationship between Dz and the particle diffusion coefficient (*D*) is the Stokes–Einstein equation, Dz = *kT*/(3πη*D*), where *k* is the Boltzmann’s constant, *T* is the temperature in Kelvin, and η is the viscosity of the medium. The equipment software algorithm was the non-negatively constrained least-squares (NNLS) for multimodal distributions.^42^ Size distributions allowed us to obtain polydispersities (*P*) related to the width of the size distribution. The zeta potential (ζ) was determined from the Smoluchowski equation ζ = μη/ε, where electrophoretic mobility is μ in 1 mM NaCl, η is the medium viscosity, and ε is the medium dielectric constant. At room temperature, Gr NPs remained stable regarding their physical properties such as size, zeta potential, and polydispersity for at least 24 h after dispersing Gr in water solution (pH 6.3–6.6).^26^

### Determination of Turbidity for Gr Dispersions in Water

Turbidity for Gr dispersions in pure water was determined as an apparent absorbance derived from the light scattered by the Gr nanoparticles in water dispersions. As a blank, the same medium of the Gr dispersion in the absence of Gr was used. Turbidity data were plotted as a function of the incident wavelength of light (Figure 3) or as a function of Gr concentration (Figure 4).

### Determination of Optical Spectra for Gramicidin in Methanol or Trifluoroethanol over the UV Region

**Figure 1:**
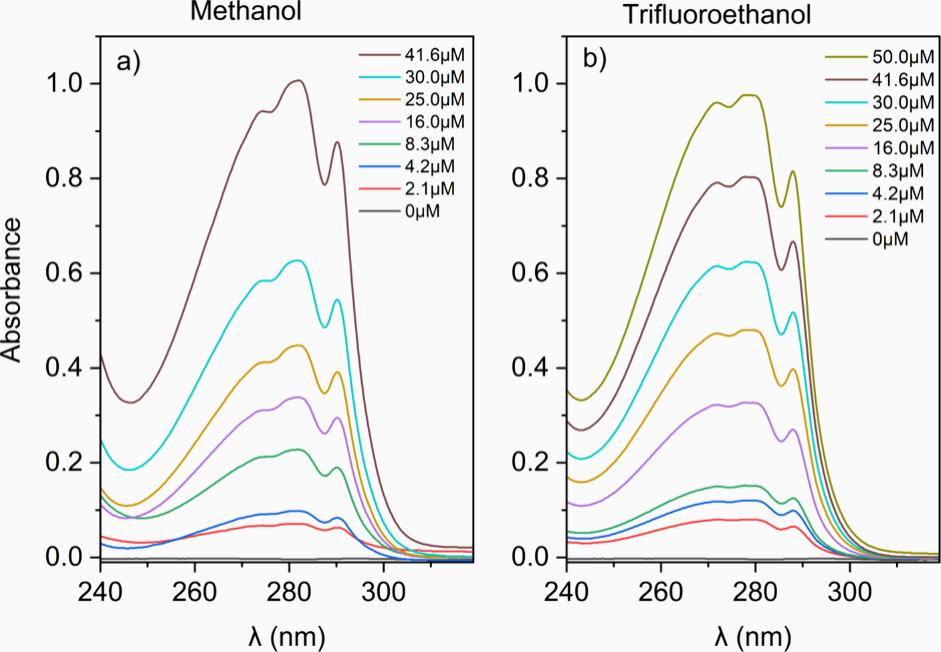
U.V. spectra for gramicidin D absorbance as a function of the wavelength of the incident light in CH_3_OH (a) or TFE (b) over a range of micromolar Gr concentrations. Absorbance at 282 nm/280 nm as a function of Gr concentration yielded linear fittings with regression coefficients (*R*) equal to 0.97924/0.99389, respectively (Supporting Information S1). The mean molar absorptivity of Gr in methanol and TFE calculated from these linear fittings was 21,340 ± 1170 and 19,100 ± 611 cm^–1^·M^–1^, respectively (Supporting Information S1).

Optical spectra for Gr in TFE or methanol were obtained at 25 °C by means of a UV–vis double-beam spectrophotometer in a 1 cm quartz cuvette with the following parameters: 0.5 nm wavelength increments; 4 s response; wavelength range of 200–360 nm; 100 nm/min scan rate; averaging 5 scans per spectrum; 10 m deg. In the blank cuvette, the same solvent for solubilizing Gr was used in the absence of Gr. Background corrections were obtained by subtracting blanks in the absence of Gr such as Gr solvent. Spectral shape was preserved by smoothing. Absorbance was plotted as a function of the wavelength of incident light over a range of Gr concentrations (Figure 1). At the wavelength of maximal absorbance, absorbances were plotted as a function of Gr concentration for determining mean molar absorptivity and its mean standard deviation from the linear fittings obtained over the 0–50 μM range of Gr concentrations (Supporting Information 1).

### Culture of Mammalian Cells and Determination of Cell Viability from Cells in Culture over a Range of Gramicidin Concentrations

The cellular lineages used to evaluate cytotoxicity of Gr nanoparticles were (1) A31,^43^ a cell line developed by Aaronson and Todaro in 1968 from disaggregated 14- to 17-day-old BALB/c embryos;^44^ (2) SVT2,^43^ a fibroblast cell from isogenic mice BALB/c; (3) the human cervix cancer cell line (HeLa); (4) L929 fibroblast cells from mouse;^45^ and (5) J774 macrophage cells from mice.^45^ All cells were obtained from the American Type Culture Collection (ATCC) and maintained at 37 °C in a humidified incubator containing 5% CO_2_. A31 and SVT2 cells were cultivated in DMEM high glucose (Gibco) supplemented with 10% fetal bovine serum, 1% HEPES (Gibco), and 2 mM l-glutamine (Gibco). For HeLa, L929, and J774, the RPMI 1640 medium (Gibco) was used, supplemented with 10% fetal bovine serum (Gibco) and 2 mM l-glutamine (Gibco), and maintained at 37 °C in 5% CO_2_. All of the cell lines were confirmed as mycoplasma free by mycoplasma PCR tests. The culture media contained 1% penicillin/streptomycin (P/S) to prevent microbial contamination and displayed pH values of 7.0–7.6.

For the cytotoxicity experiments, gramicidin stock dispersions in isotonic d-glucose 0.264 M were prepared at 5, 10, 20, 30, 40, and 50 μM Gr and 10 μL of each dispersion was added to 90 μL of the culture medium. One should notice that the final TFE concentration will be 0.1% in volume. The cytotoxicity of gramicidin nanoparticles was evaluated against all five cell lines. The cells were seeded at 1.5 × 10^4^ cells per well in 96-well microplates. After 24 h, a new medium containing gramicidin (0.5, 1.0, 2.0, 3.0, 4.0, and 5.0 μM Gr) or medium only, or 90 μL medium and 10 μL d-glucose 0.264 M (negative controls), or 1% Triton X-100 (Cayman), as a positive control, were added and incubated at 37 °C and 5% CO_2_ for 24 h.

The 3-(4,5-dimethylthiazol-2-yl)-2,5-diphenyl-2*H*-tetrazolium bromide (MTT) colorimetric assay was performed for quantitative determination of cell viability.^46^ The MTT assay is based on the intracellular reducing power of dehydrogenases and reducing agents of living cells. Formazan, a violet-blue, water-insoluble product of the reduction, accumulates mainly in hydrophobic cytoplasmic domains and can be solubilized using appropriate solvents. Cells were incubated with MTT (3-(4,5-dimethylthiazol-2-yl)-2,5-diphenyltetrazolium bromide, Sigma) at 5 mg/mL and 10 μL/well for 2 h at 37 °C. Thereafter, the medium was withdrawn, and 100 μL/well of PBS containing isopropanol and 0.01 M HCl was added for solubilizing formazan. The absorbance was determined using a multiwell scanning spectrophotometer (SpectraMax, USA) at 570 nm. The cell viability was plotted as column graphics, and the IC50 value for cytotoxicity was taken as the concentration of gramicidin where cell viability is 50% (IC50). The IC50 was calculated from the concentration–response curve using GraphPad Prism. The minimal microbicidal concentrations (MMCs) against microbes taken from the literature^19,47^ were included in Table 1 for comparison with IC50 values for mammalian cells.

**Table 1:** Differential Cytotoxicity of Gr NPs against Microbes and Mammalian Cells^a^

**cells**	**cell line**	**[Gr]/μM IC50**	**[Gr]/μM MMC**
macrophages/10^4^ cells	J774	3.3 (0.3)	
fibroblasts/10^4^ cells	L929	>5.0 (0.3)	
	A31	2.5 (0.3)	
	SVT2	1.7 (0.3)	
HeLa/10^4^ cells	HeLa	3.5 (0.3)	
*C. albicans***/**10^7^ CFU			2.0 (7)^b^
*E. coli***/**10^8^ CFU			5.0 (0.3)^c^
*S. aureus*/10^7^ CFU			20.0 (7)^b^

a The reduction in cell viability is expressed as the number of logarithmic cycles (in between parentheses) besides each minimal microbicidal concentration (MMC) or concentration for killing 50% of the 10^4^ mammalian cells (IC50).

b One asterisk refers to data from Pérez-Betancourt et al.^19^

c Data from Ragioto et al.^47^ One should notice that for killing 50% of a mammalian cell line, the number of logarithmic cycles corresponding to this reduction of cell viability is 0.3.

### Statistical Analysis

Experiments were carried out in quadruplicate by assay and the experimental duplicate made in two different days, in a total of eight replicates by each cell and by each gramicidin concentration. The results were expressed as mean ± SEM. Differences between treatments and controls were analyzed by the Student’s *t* test (unpaired or nonparametric test, assuming normal Gaussian distributions); all tests were followed by the Bonferroni post-test, using the software GraphPad Prism (GraphPad Software, Inc., San Diego, USA). Differences between groups were considered statistically significant at *p < 0.5, **p = 0.005, ***p < 0.001, and ****p < 0.0001.

## Results and Discussion

### Analytical Determination of Gr Concentration from Light Absorption

In order to ascertain the analytical Gr concentration in the stock solution to be used to prepare the colloidal dispersions of Gr in water, light absorption spectra of Gr molecules solubilized in two different solvents (methanol and trifluoroethanol) over a range of Gr concentrations were obtained (Figure 1).

As shown in Figure 1, absorption maxima at 282 nm in methanol and at 280 nm in TFE have been ascribed to light absorption by Gr tryptophans and often used in the literature to quantify the Gr concentration.^48−50^ Killian and co-workers reported 20,700 cm^–1^ M^–1^ as the molar extinction coefficient for Gr at 280 nm in methanol.^48^ The light absorption by the four Gr tryptophan residues in each Gr molecule originates the intrinsic Gr tryptophan′s fluorescence emission. Natural gramicidin D (Gr) has ∼85% of gramicidin A, which has four tryptophan residues at positions 9, 11, 13, and 15; intrinsic fluorescence is due to the tryptophan residues.^51^

### The Self-Assembly of Gramicidin D in Water as Spherical Nanoparticles and Their Compliance with Rayleigh Law for the Light Scattering by Spherical Nanoparticles

In order to evaluate the reproducibility of the self-assembly of Gr in water, several equivalent dispersions of Gr prepared from the same stock solution in TFE were obtained and their physical properties evaluated from photographs, turbidity, DLS, and compliance of the experimental results with predictions from Lord Rayleigh law for the light scattered by spherical particles with dimensions much smaller than the wavelength of the incident light (λ).^52,53^
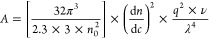


In the above equation by Lord Rayleigh, the scatter-derived absorbance *A* (turbidity) of a dispersion of particles, which are much smaller than the wavelength of incident light, is proportional to the concentration of particles ν, to the square of the anhydrous mass of the particle *q*, and inversely related to the fourth power of the wavelength of light λ incident on the sample.

**Figure 2:**
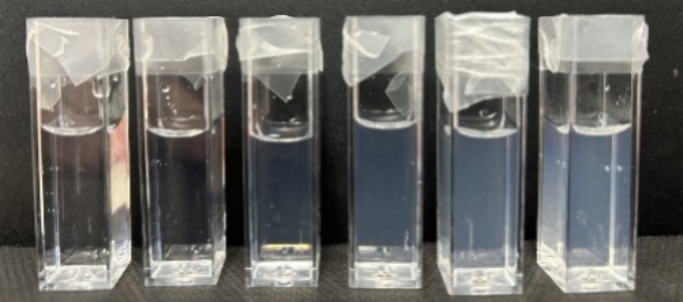
Photos of Gr colloidal dispersions in water at 5, 10, 20, 30, 40, and 50 μM Gr.

Figure 2 shows photographs of Gr colloidal dispersions in water over a range of micromolar Gr concentrations (5, 10, 20, 30, 40, and 50 μM). The milky aspect is due to the light being scattered by Gr nanoparticles (NPs) in the dispersions. Indeed, the Gr NPs were previously reported by our group as the preferred outcome of Gr self-assembly in aqueous solutions over the micromolar range of Gr concentrations, as visualized from scanning electron micrographs.^19^ At room temperature, Gr NPs remained stable regarding their physical properties such as size, zeta potential, and polydispersity for at least 24 h after dispersing Gr in water solution.^26^

For amphiphilic compounds able to self-assemble as micelles in water solution, the critical micelle concentration (CMC) is a valuable parameter depending on the magnitude of intermolecular interaction forces and can be determined experimentally.^54^ On Figure 2, one should consider that turbidity is not sufficiently accurate at 400 nm for determining the CMC for Gr. Using DLS, previous experimental data from our lab showed that the CMC for Gr should be lower than 0.005 mM, which is the lowest Gr concentration where Gr NPs could be detected.^19^ In addition, the conductance for Gr NPs in water (about 8 μS) is very low over the whole range of concentrations tested (0.0005–0.050 mM) and cannot be used to determine CMC. Due to hydrophobic lateral groups in most amino acid residues, Gr has basically a hydrophobic nature facilitating its self-assembly in water solution driven by the hydrophobic effect. Gr is a hydrophobic and neutral antimicrobial peptide for which CMC determination is difficult, as compared with surfactants and detergents with more significant polar or charged moieties.

**Figure 3:**
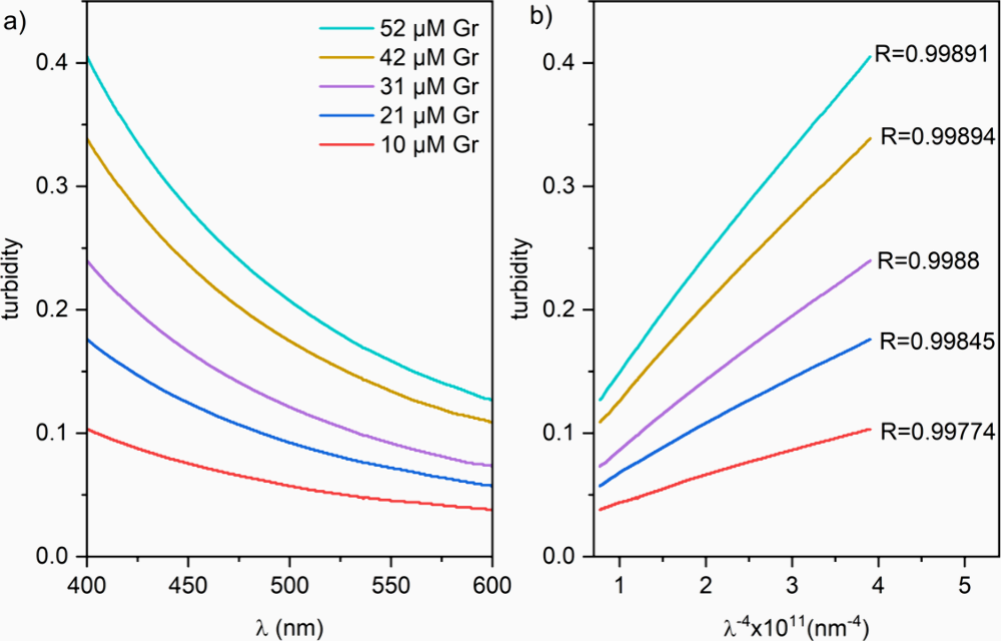
Turbidity for Gr colloidal dispersions in pure water as a function of the wavelength of incident light (λ) (a) or as a function of λ^–4^ (b).

Turbidity or scatter-derived absorbance dependence on the wavelength of the incident light for Gr colloidal dispersions in water over a range of micromolar Gr concentrations is shown in Figure 3a. There was a linear dependence of the turbidity on 1/λ^4^ (Figure 3b). The linear regression coefficients were high (Figure 3b), suggesting excellent agreement between turbidity experimental data and Rayleigh predictions for spherical NPs with sizes much smaller than the wavelength of the incident light. In fact, the nanometric sizes around 150 nm (mean hydrodynamic diameter) for the Gr NPs were previously reported by our group.^19,26,27^ Here, we show the spherical shape of the NPs from the agreement between the Rayleigh predictions and the experimental data (Figure 3).

**Figure 4:**
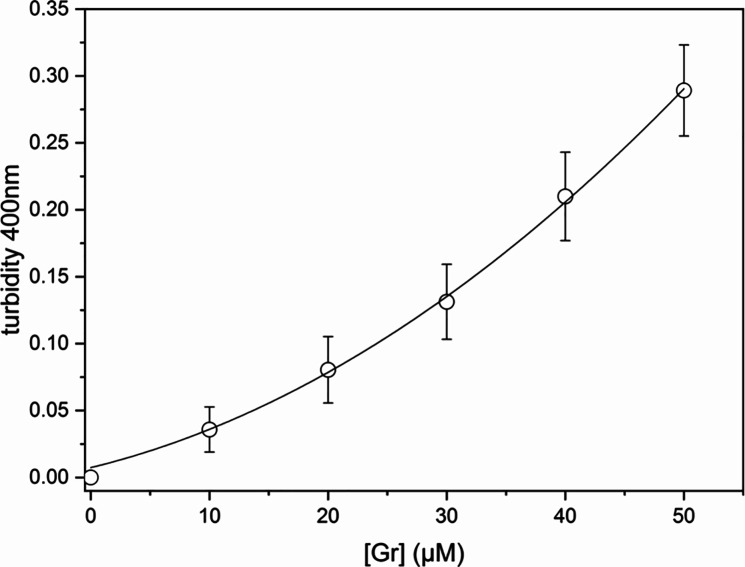
Turbidity at 400 nm for Gr colloidal dispersions in pure water as a function of [Gr].

**Figure 5:**
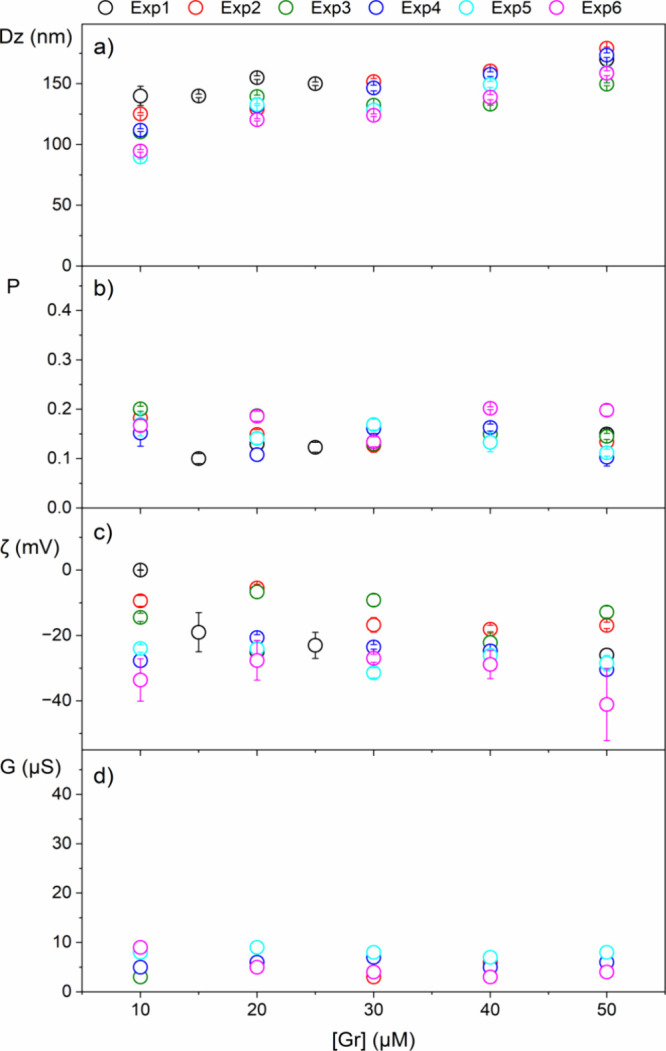
Reproducibility of self-assembled gramicidin D (Gr) dispersions in water over a micromolar range of Gr concentrations for five different experiments from determinations of physical properties such as mean hydrodynamic diameter Dz (a), polydispersity *P* (b), zeta potential ζ (c), and conductance *G* (d). Each dispersion contains 1% in volume of the solvent trifluoroethanol (TFE).

As shown in Figure 4, five equivalent experiments allowed calculation of the mean turbidity at 400 nm and their mean standard deviations as a function of Gr concentration. The mean turbidity values displayed a quadratic dependence on the Gr concentration. The increase in turbidity at 400 nm with the Gr concentration could be explained by the following alternative or simultaneous possibilities: (1) an increase in particle size with the Gr concentration, (2) an increase in particle number density with the Gr concentration, or (3) both. Experimental DLS data showed a slight increase in the particle size with the Gr concentration (Figure 5a). The Rayleigh equation predicts that turbidity of the Gr NP dispersions depends on the square of the anhydrous mass of the NPs and only on the first power of the particle number density (particle concentration). This means that the anhydrous mass of NPs is more important than the NP concentration for determining turbidity. Thus, one may conclude that the increase in turbidity with Gr concentration is probably due to the increase in mean anhydrous mass of the NPs.

From five independent experiments, the reproducible character of the physical properties of Gr dispersions in water is shown in Figure 5. As observed in Figure 5a, the particle size increased slightly with the Gr concentration (0–50 μM), in agreement with previously reported data for a single experiment.^19^ In Figure 5b, the polydispersity (*P*) for the colloidal dispersions of Gr exhibited relatively low values (0.10–0.20) over the entire range of Gr concentrations tested. NPs in the dispersions were homogeneous in size, as reported from scanning electron micrographs (SEM) previously.^19^ As observed in Figure 5c, the negative zeta potential determined at the shear plane of the Gr NPs might be related to anisotropy of electrostatic potential distribution on the Gr molecule, corroborating the molecular dynamics simulation for Gr reported by Chen and Wei.^55^ The primary structure of Gr would suggest neutral character for the molecule due to its net charge equal to zero.

As shown in Figure 5d, the very low conductance (*G*) for the Gr dispersions was not affected by Gr concentration, in agreement with previously reported data.^19^*G* values were also very reproducible for different experiments.

### Cytotoxicity of Gr Nanoparticles against Mammalian and Microbial Cells

The Supporting Information shows detailed data for mammalian cell viability in the presence of the Gr NPs over the 0–5 μM range of Gr concentrations. Five different mammalian cell lines in culture responded similarly to the interaction with Gr NPs, as depicted from the similar values of IC50 shown in Table 1.

One should notice that IC50 corresponds to the Gr dose provided by the Gr NPs that kills 50% of the total number of interacting cells. Since the mammalian cell count was 10,000 cells per well, killing 50% means killing 5000 cells at the Gr dose corresponding to IC50. The differential cytotoxicity of the Gr NPs is presented in Table 1, where similar values for IC50 for mammalian cells and MMC for microbes are observed.

At IC50, micromolar doses of Gr killed 5000 mammalian cells, leaving 5000 cells alive. At the MMC equal to 2 μM Gr, 10^7^*Candida albicans* cells were killed, leaving no cells alive. Thus, differential cytotoxicity indeed takes place. However, the literature on the Gr mechanism of action has consistently revealed that Gr acts via the same mechanism in microbial or mammalian cell membranes, namely, changing the semipermeable nature of these membranes and preventing their function as a natural barrier against ions. In natural cell membranes, specific activity of energy-dependent transporters such as Na^+^/K^+^ ATPases keep the ionic balance in vivo. Apparently, the Gr channels dissipate ion concentration gradients across cell membranes, following similar mechanisms in microbial or mammalian cell membranes.

The remarkable performance of the Gr NPs against *C. albicans* killing seven logarithmic cycles at 2 μM Gr was previously reported for Gr NPs in aqueous colloidal dispersions.^19^ Gr NP dispersions were also casted and dried on surfaces to coat both hydrophobic and hydrophilic materials of biomedical importance.^26,27^ In combination with the antimicrobial, cationic, and hydrophilic polymer poly(diallyldimethylammonium) bromide,^32−34,56,57^ Gr NPs formulated as dispersions in water or as coatings yielded transient but very efficient, broad-spectrum antimicrobial protection.^8,9^

Against *S. aureus*, Gr NPs induced complete loss of cell viability killing seven logarithmic cycles of the bacteria only at relatively high doses (about 20 μM Gr). This was the largest dose of Gr required to kill cells (Table 1).

Curiously, Gr doses for viability of different prokaryotic or eukaryotic cells did not differ substantially, as shown in Table 1, suggesting that all cells were submitted to a similar mechanism of loss of cell viability, namely, insertion of the Gr molecules in the cell membrane, formation of the dimeric channel for single-file diffusion of cations, and impaired ionic balance between the intracellular and extracellular compartments leading to cell disruption and death.

The IC50 doses for Gr were also very similar for normal and cancerous cells. This might have been because gramicidin D is a neutral peptide that depends only on the hydrophobic effect to become inserted in the host cell membrane. Thereby, the higher proportion of negatively charged phosphatidylserine reported in cancer cells as compared to normal cells considered an asset to improving effects of cationic AMPs^58^ would not affect gramicidin D insertion in the cell membrane mostly driven by the hydrophobic effect.

In vivo, cells actively maintain a high concentration of K^+^ and low concentration of Na^+^. Gr inserted in cell membranes allows single-file diffusion of ions in accordance with the concentration gradient. In model membranes such as lipid bilayer vesicles, Gr dimeric channels promoted diffusion of solutes such as cations and small neutral molecules, which did not occur in control cells in the absence of Gr.^47,50^ In cells, Gr channels also became inserted in the inner mitochondrial membrane promoting dissipation of the protons gradient and hampering ATP biosynthesis, thereby causing energy depletion, metabolic dysfunction, and cell death, a most valuable asset against renal cell carcinoma.^38^

## Conclusions

In conclusion, antimicrobial nanoscale materials are frequently constructed by molecular self-assembly leading to significant progress against antimicrobial resistance.^25^ As alternatives to antibiotics, antimicrobial peptides (AMPs) can display potent activity against a wide range of microbes and are unable to induce the appearance of resistance. However, AMP degradation in vivo is a major drawback, which can be dealt with by natural or induced self-assembly for improving their bioavailability.^59^ Here, we show that gramicidin, a channel model of membrane proteins,^60^ is endowed with the interesting property of natural self-assembly in water dispersions to form spherical and reproducible NPs that scatter the incident light in accordance with Lord Rayleigh predictions and kill both mammalian and microbial cells over a similar range of micromolar concentrations. Although this looks like a disadvantage, one should recall that local injection of Gr NPs in cancers could become an important asset to induce immunogenic cell death and turn a non-responsive tumor to immunotherapy into a responsive one.^61^ Immunogenic cell death (ICD) triggered by antimicrobial lytic peptides such as mellitin^62,63^ or linear gramicidins has had its anticancer effects often reported.^64^ Tumor cell lysis releases tumor-specific antigens able to elicit ICD in situ and systemically. The Gr NPs described in the present work may evolve to provide not only broad-spectrum antimicrobial dispersions and coatings in appropriate combinations with cationic polymers but also local and systemic treatments against cancer.

## Supporting Information Available

(S1) Linear dependence of gramicidin D light absorption on gramicidin D concentration yielding mean molar absorptivity for gramicidin D in methanol or trifluoroethanol ± mean standard deviation; (S2) photographs from optical microscopy for mammalian cells in the presence or in the absence of the gramicidin nanoparticles over a range of Gr concentrations; and (S3) cell viability curves for mammalian cells as a function of gramicidin concentration for the five cell lineages tested (PDF)
