# Supplementary material for: Colloidal Dispersions of Gramicidin D in Water: Preparation, Characterization, and Differential Cytotoxicity
Source: ACS Omega. 2025 Feb 24;10(8):8611–8. doi: 10.1021/acsomega.4c11133 (PMC11886900; doi:10.1021/acsomega.4c11133)
Supplement: Supplementary file 1 — ao4c11133_si_001.pdf [file ao4c11133_si_001.pdf]

Colloidal dispersions of gramicidin D in water: preparation, characterization and differential cytotoxicity

*Ricardo Márcio-e-Silva[1], Bianca R. Bazan[1], Rodrigo T. Ribeiro[1], Sarah N. C. Gimenes[2], Bianca C. L. F. Távora[2], Eliana L. Faquim-Mauro[2] and Ana M. Carmona-Ribeiro [1,] **

[1] Biocolloids Laboratory, Departamento de Bioquímica, Instituto de Química, Universidade de São Paulo, Avenida Professor Lineu Prestes, 748, Butantan, São Paulo SP 05508-000, Brazil. E-mail: mcribeir@iq.usp.br; Tel: +55 11 30912164

[2] Immunopathology Laboratory, Butantan Institute, Av. Vital Brasil, 1500, São Paulo 05503-900, Brazil

*Author to whom correspondence should be addressed: mcribeir@iq.usp.br

KEYWORDS: self-assembly of gramicidin D in water; nanoparticles; natural antimicrobial peptide; cell viability of mammalian cells in culture; turbidimetry and Rayleigh Law; light absorption and scattering by gramicidin D and/or its assemblies.

S1. Linear dependence of gramicidin D light absorption on gramicidin D concentration yielding mean molar absorptivity for gramicidin D in methanol or trifluoroethanol ± mean standard deviation

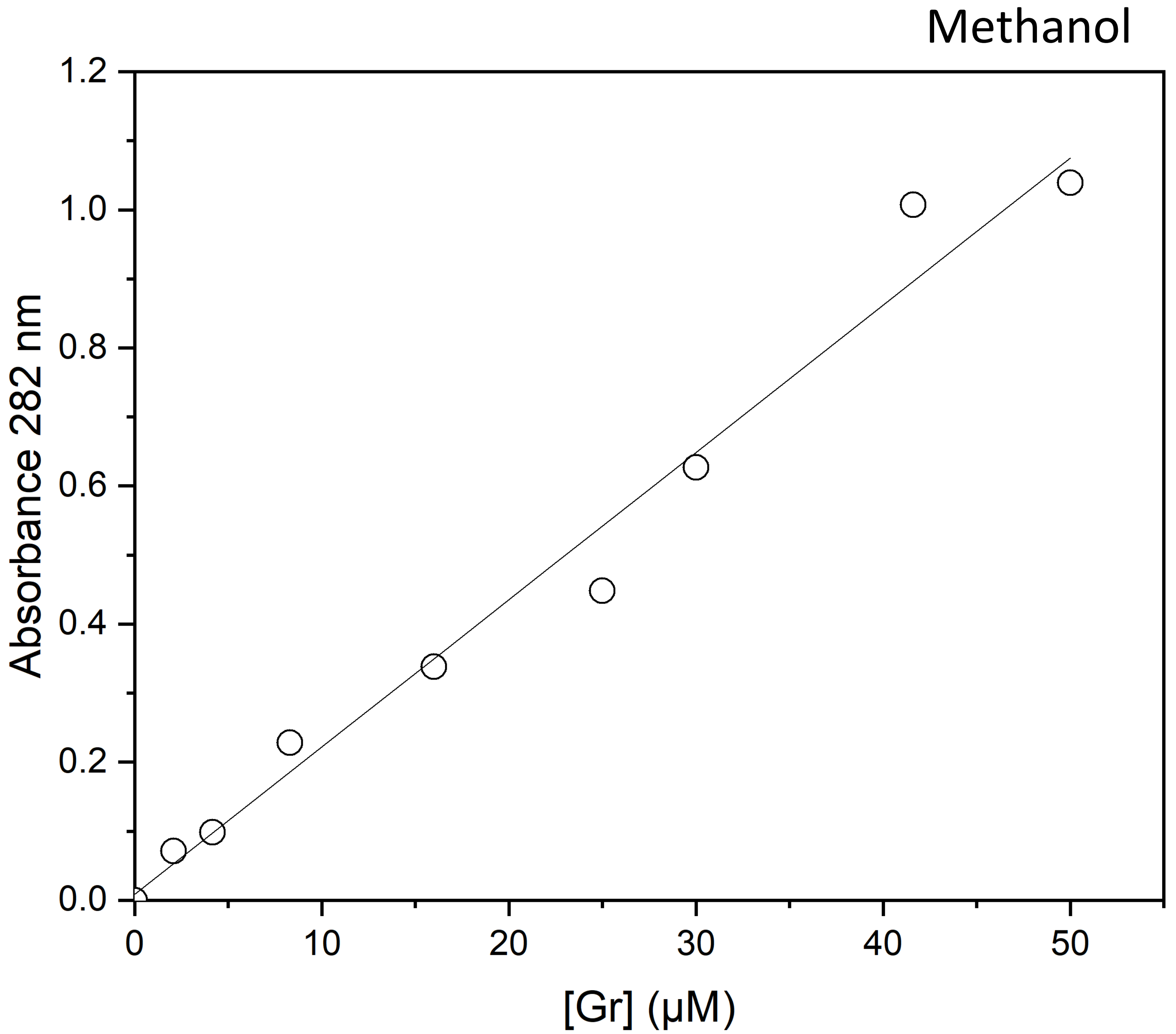

Equation	y = a + b*x
Plot	Absorbance 282 nm
Weight	No Weighting
Intercept	0.00844 ± 0.03057
Slope	0.02134 ± 0.00117
Residual Sum of Squares	0.02524
Pearson's r	0.98957
R-Square (COD)	0.97924
Adj. R-Square	0.97628

Trifluoroethanol

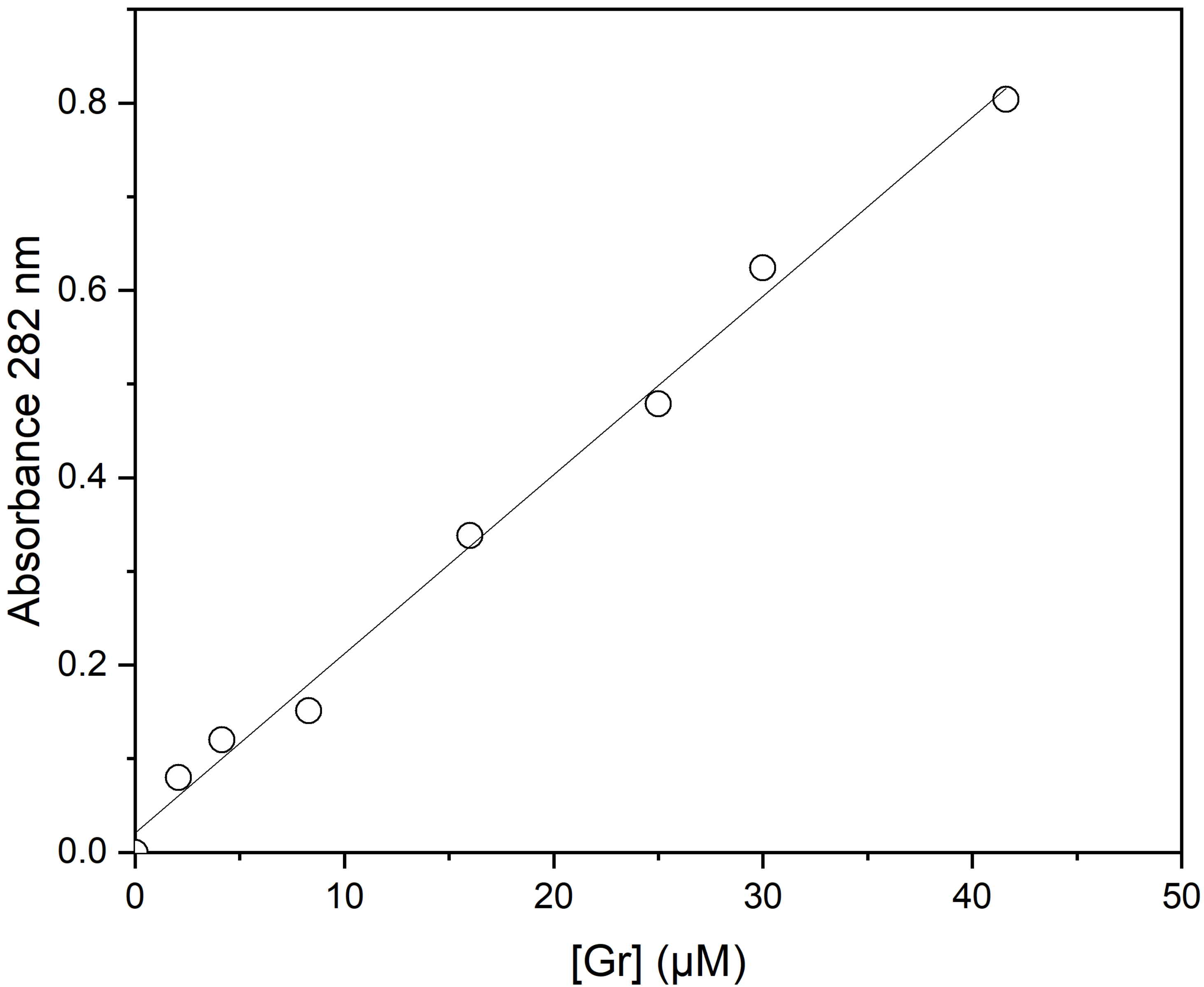

Equation	y = a + b*x
Plot	Absorbance 282 nm
Weight	No Weighting
Intercept	0.02089 ± 0.01298
Slope	0.0191 ± 6.1166E-4
Residual Sum of Squares	0.00355
Pearson's r	0.99694
R-Square (COD)	0.99389
Adj. R-Square	0.99287

S2. Photographs from optical microscopy for mammalian cells in the presence or in the absence of the gramicidin nanoparticles over a range of Gr concentrations

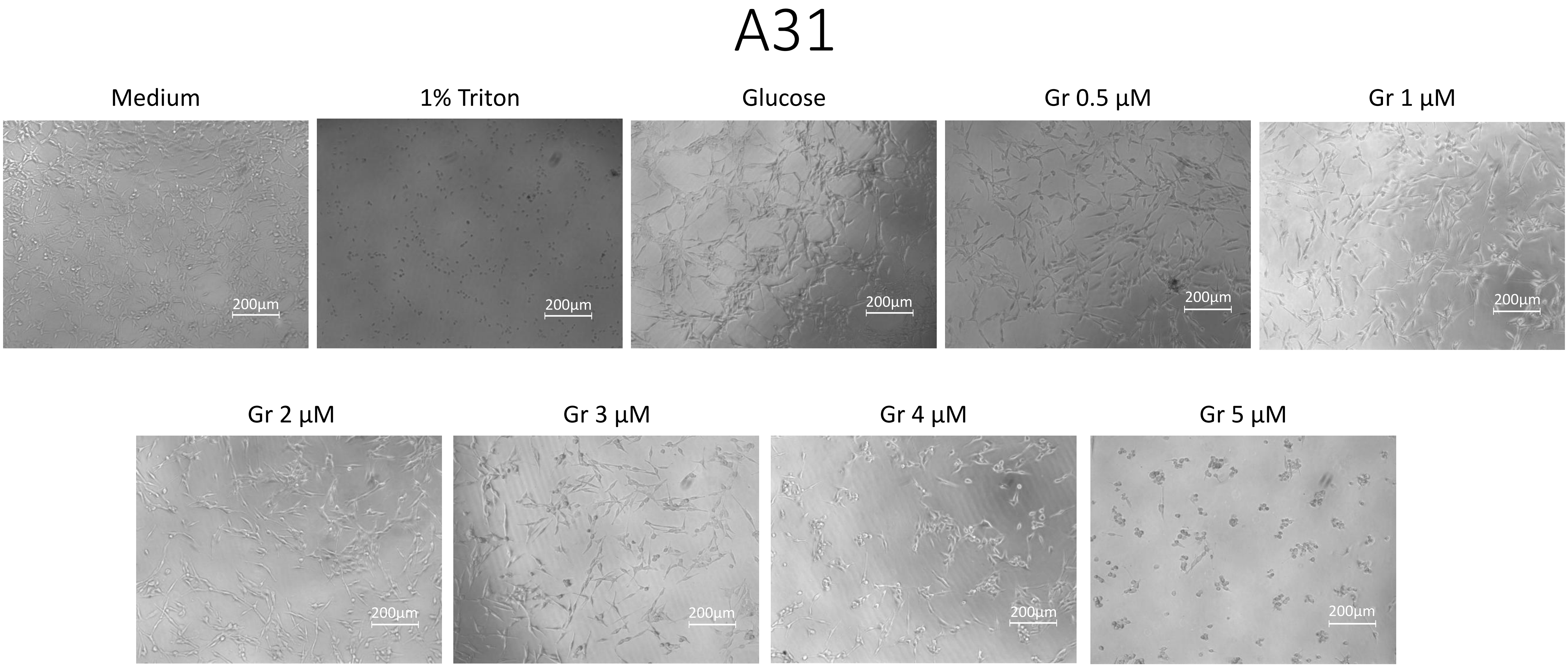

SVT2

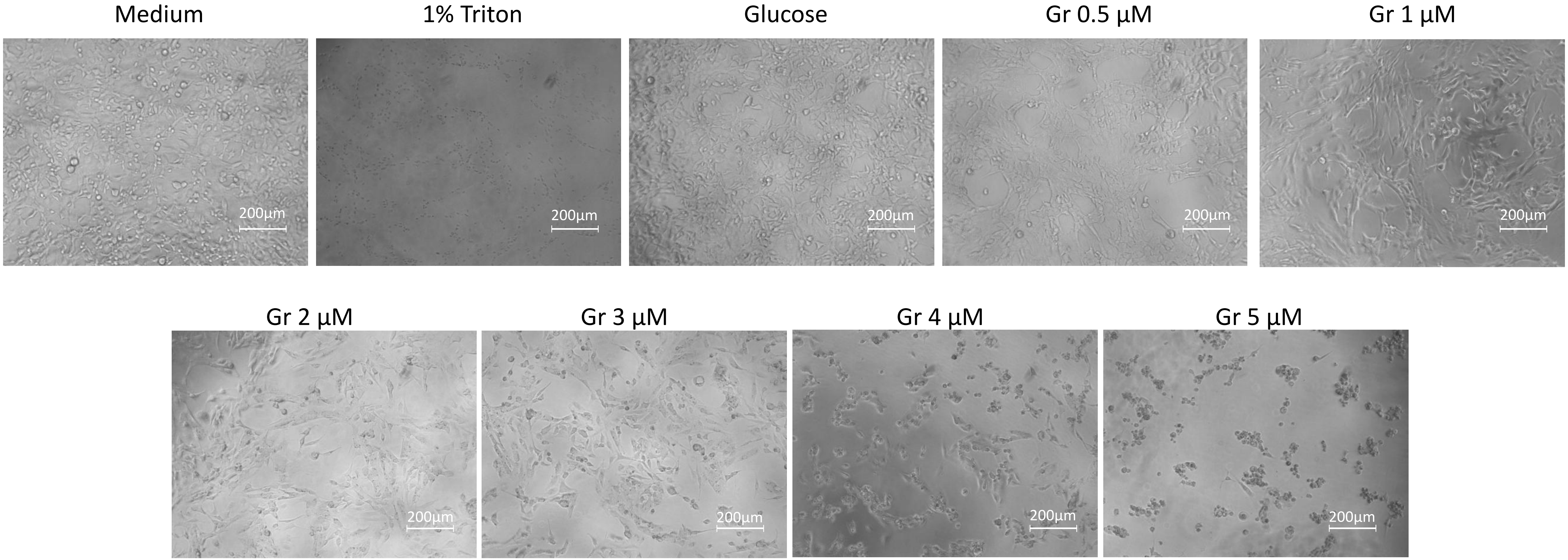
Medium
1% Triton
Glucose
Gr 0.5 µM
Gr 1 µM
Gr 2 µM
Gr 3 µM
Gr 4 µM
Gr 5 µM
200µm

L929

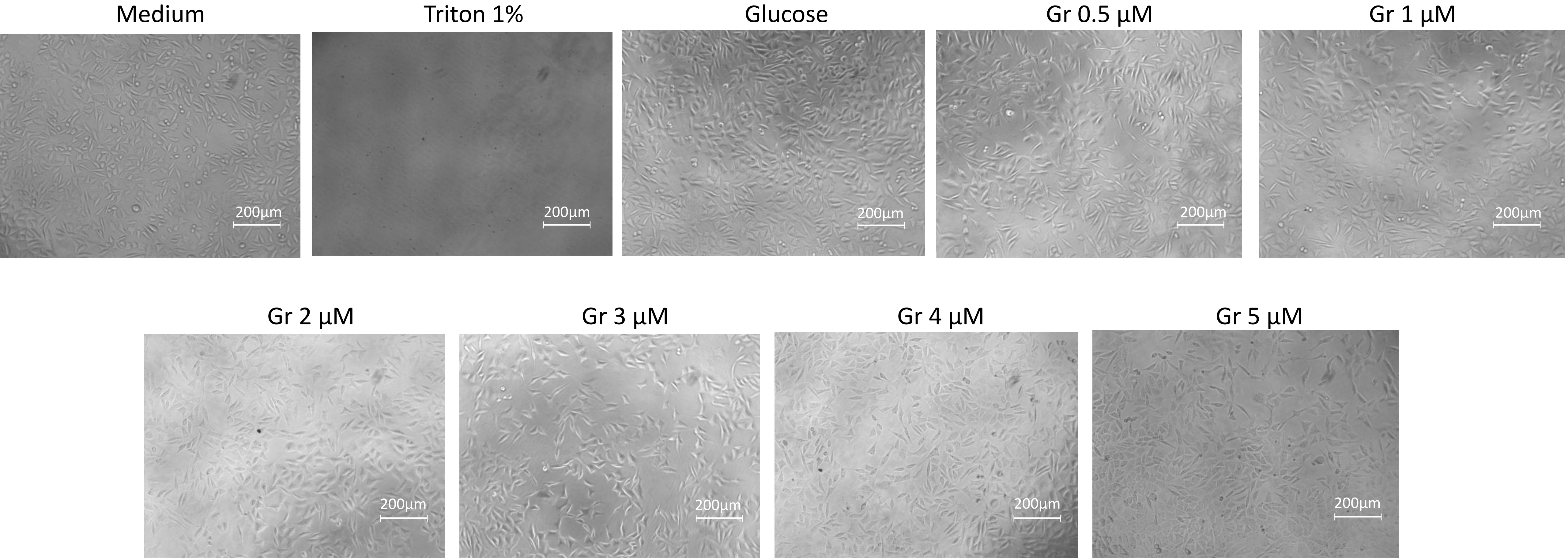
Medium
Triton 1%
Glucose
Gr 0.5 µM
Gr 1 µM
Gr 2 µM
Gr 3 µM
Gr 4 µM
Gr 5 µM
200µm

J774

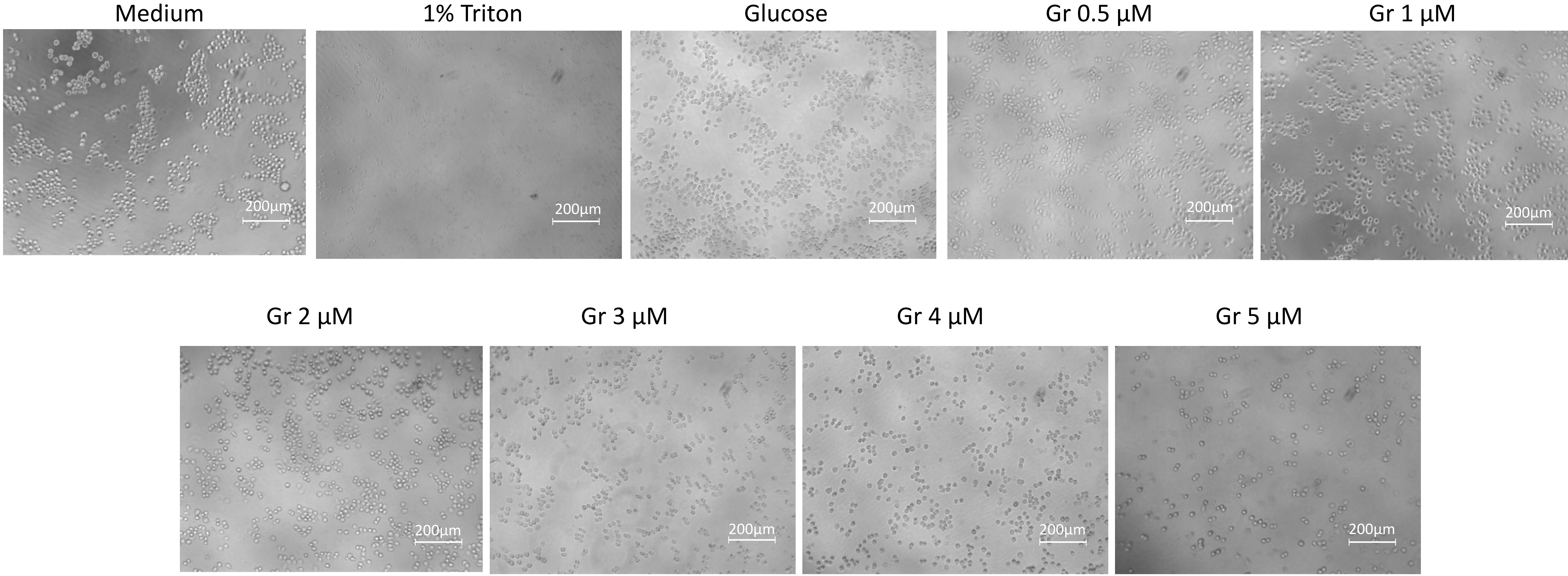
Medium
1% Triton
Glucose
Gr 0.5 µM
Gr 1 µM
Gr 2 µM
Gr 3 µM
Gr 4 µM
Gr 5 µM
200µm

HeLa

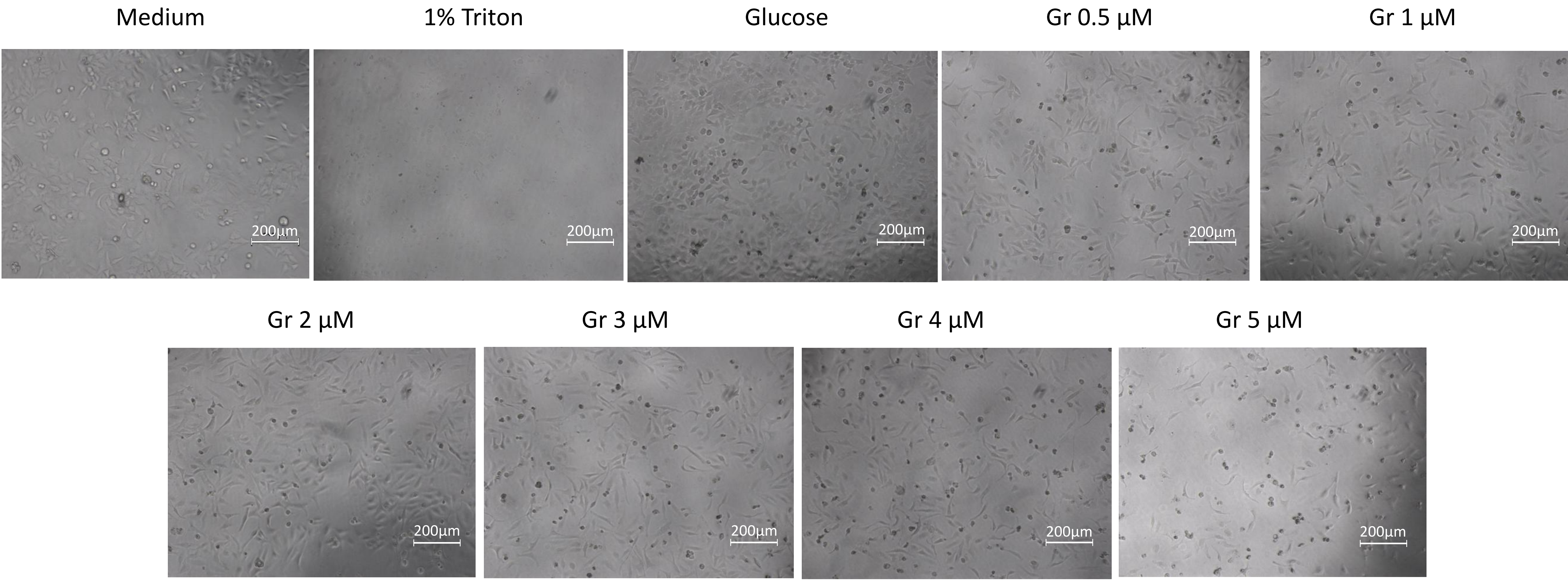
Medium
1% Triton
Glucose
Gr 0.5 µM
Gr 1 µM
Gr 2 µM
Gr 3 µM
Gr 4 µM
Gr 5 µM
200µm

S3. Cell viability curves for mammalian cells as a function of gramicidin concentration for the five cell lineages tested

A31

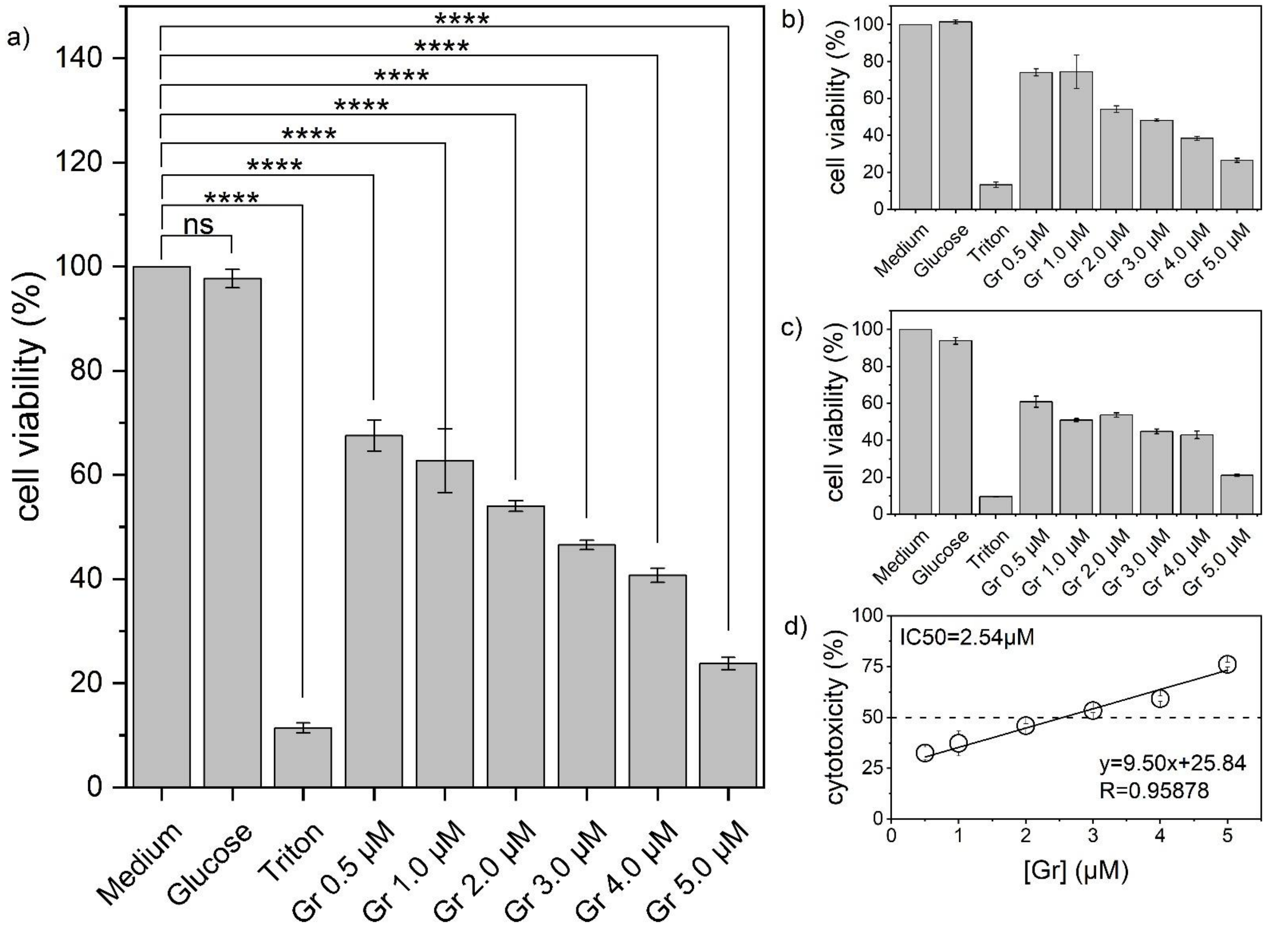

HeLa

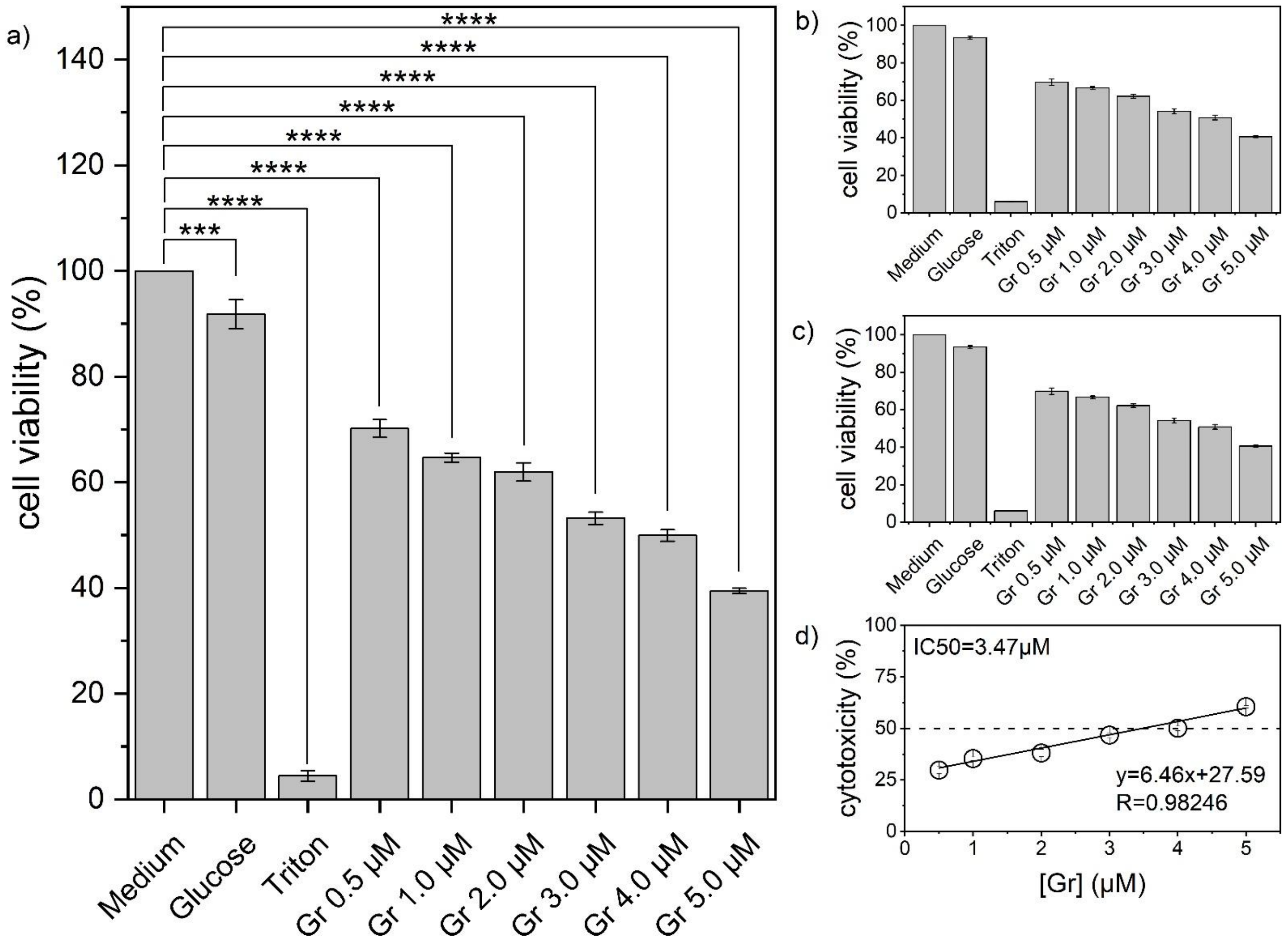

a)
cell viability (%)
****
***
Medium
Glucose
Triton
Gr 0.5 µM
Gr 1.0 µM
Gr 2.0 µM
Gr 3.0 µM
Gr 4.0 µM
Gr 5.0 µM
b)
c)
d)
IC50=3.47µM
cytotoxicity (%)
y=6.46x+27.59
R=0.98246
[Gr] (µM)

J774

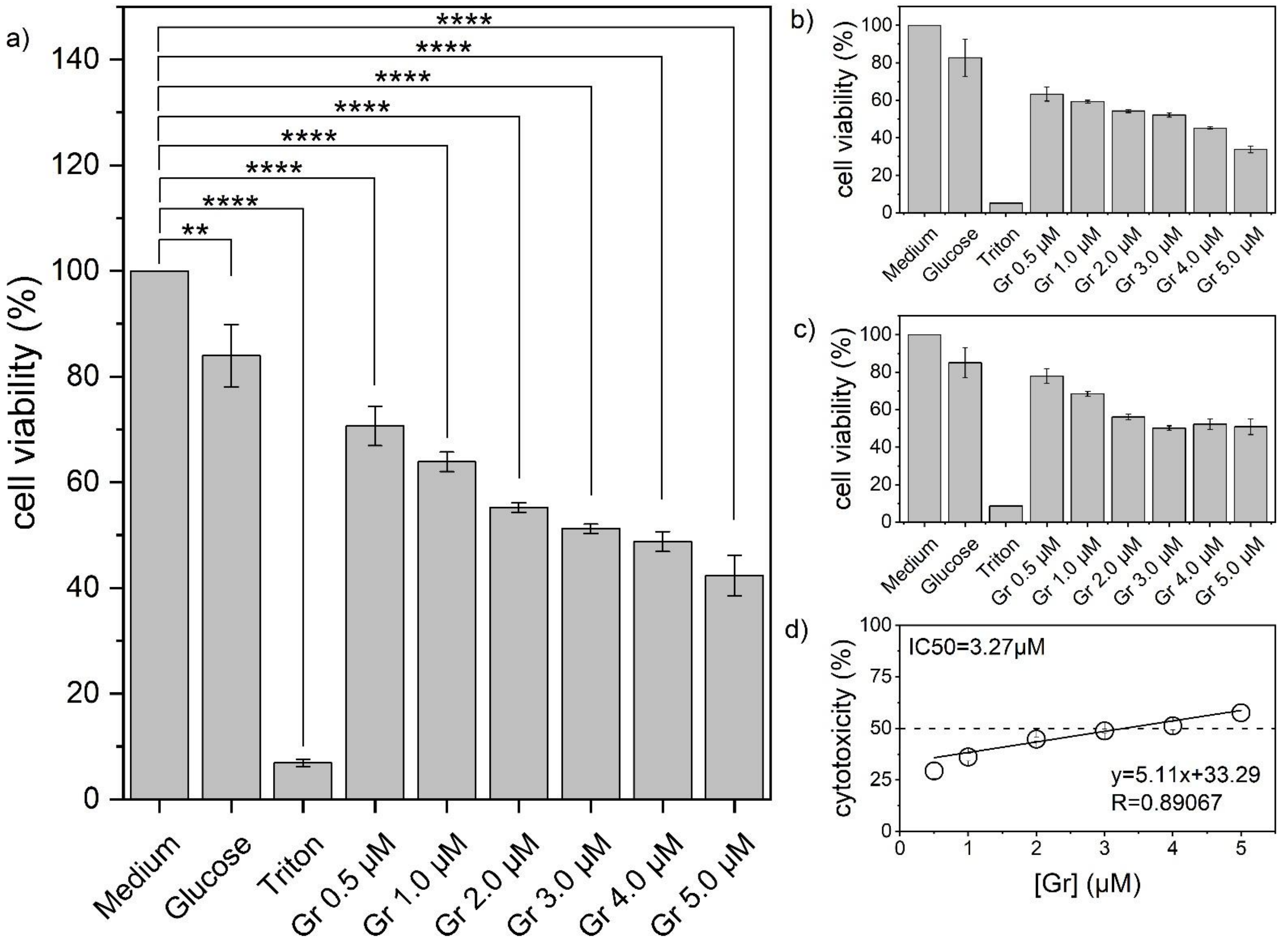

a)
cell viability (%)
****
**
Medium
Glucose
Triton
Gr 0.5 μM
Gr 1.0 μM
Gr 2.0 μM
Gr 3.0 μM
Gr 4.0 μM
Gr 5.0 μM
b)
c)
d)
cytotoxicity (%)
IC50=3.27μM
y=5.11x+33.29
R=0.89067
[Gr] (μM)

L929

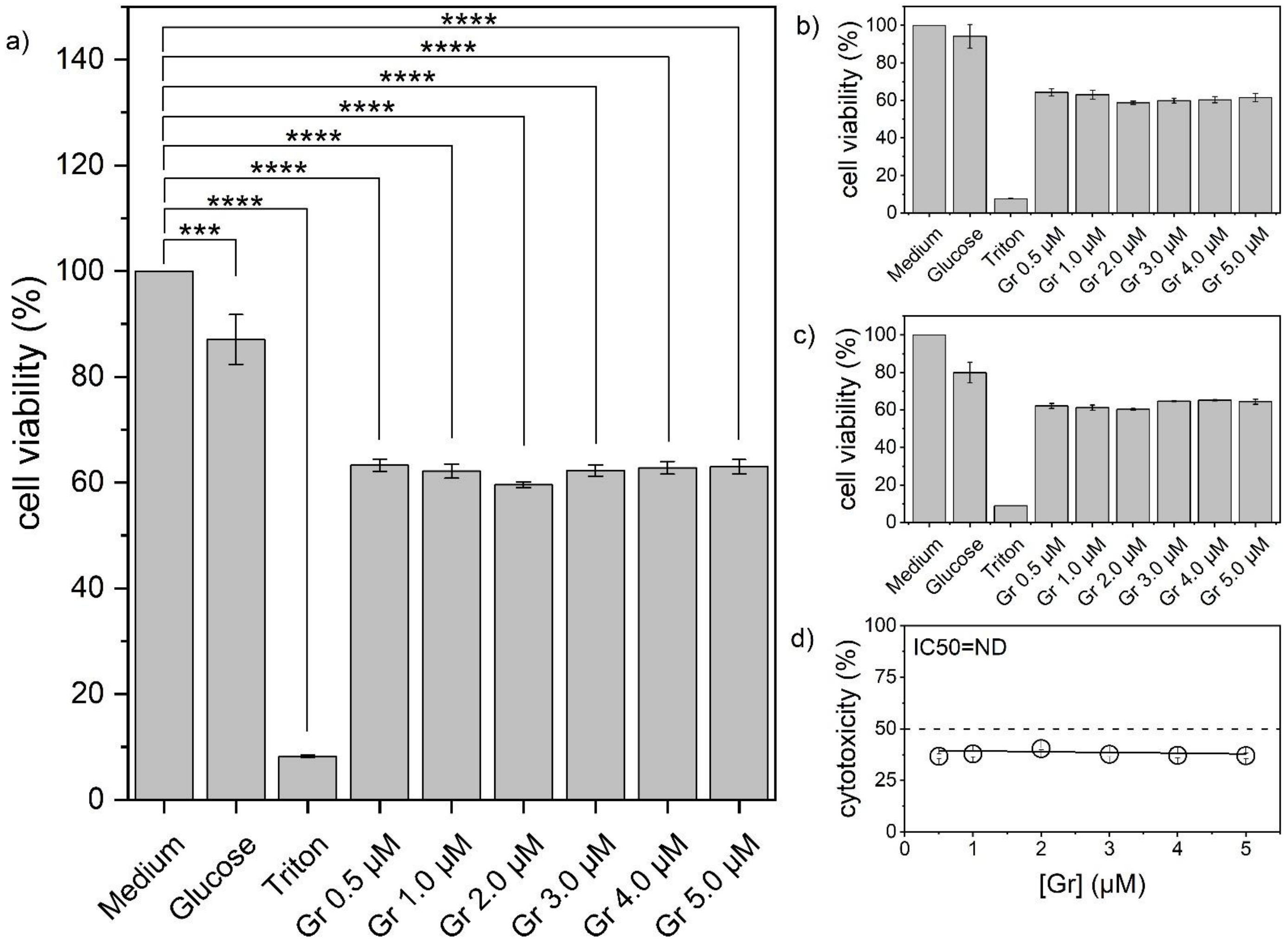
a)
b)
c)
d)
cell viability (%)
cytotoxicity (%)
IC50=ND
[Gr] (μM)
Medium
Glucose
Triton
Gr 0.5 μM
Gr 1.0 μM
Gr 2.0 μM
Gr 3.0 μM
Gr 4.0 μM
Gr 5.0 μM
***
****

SVT2

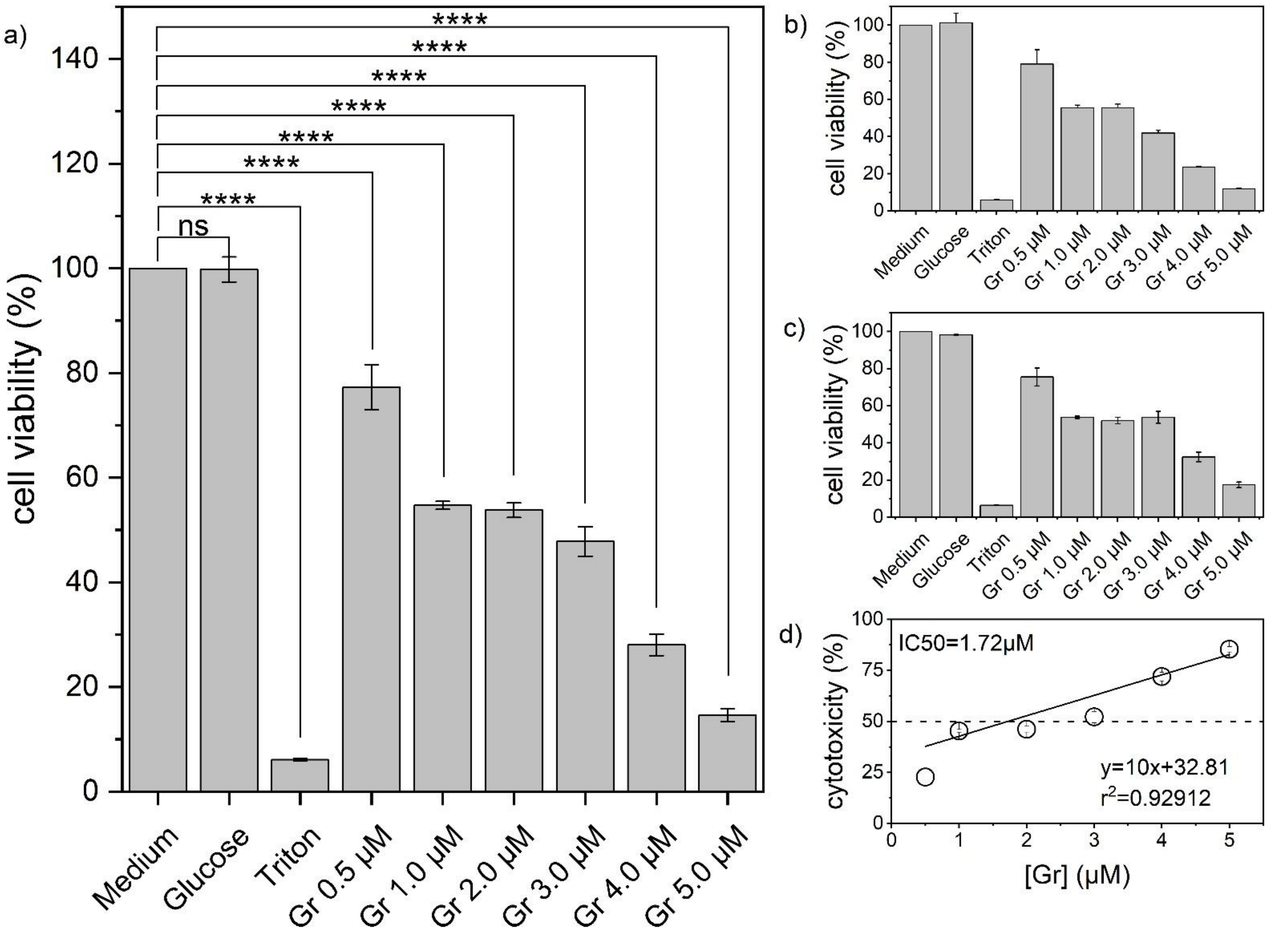

a)
cell viability (%)
ns
****
Medium
Glucose
Triton
Gr 0.5 µM
Gr 1.0 µM
Gr 2.0 µM
Gr 3.0 µM
Gr 4.0 µM
Gr 5.0 µM
b)
c)
d)
cytotoxicity (%)
IC50=1.72µM
y=10x+32.81
r2=0.92912
[Gr] (µM)
